# Knockdown of Peroxiredoxin V increased the cytotoxicity of non-thermal plasma-treated culture medium to A549 cells

**DOI:** 10.18632/aging.204063

**Published:** 2022-05-11

**Authors:** Hu-Nan Sun, Xiao-Yu Guo, Dan-Ping Xie, Xiao-Ming Wang, Chen-Xi Ren, Ying-Hao Han, Nan-Nan Yu, Yu-Lan Huang, Taeho Kwon

**Affiliations:** 1 Stem Cell and Regenerative Biology Laboratory, College of Life Science and Biotechnology, Heilongjiang Bayi Agricultural University, Daqing 163319, Heilongjiang, China; 2 Yabian Academy of Agricultural Science, Longjing 1334000, Jilin, China; 3 Primate Resources Center, Korea Research Institute of Bioscience and Biotechnology (KRIBB), Jeongeup-si 56216, Jeonbuk, Republic of Korea

**Keywords:** Peroxiredoxin V, non-thermal plasma-activated medium, lung cancer, cytotoxicity, mitogen-activated protein kinase

## Abstract

Administration of non-thermal plasma therapy via the use of plasma-activated medium (PAM) might be a novel strategy for cancer treatment, as it induces apoptosis by increasing reactive oxygen species (ROS) levels. Peroxiredoxin V (PRDX5) scavenges ROS and reactive nitrogen species and is known to regulate several physiological and pathological reactions. However, its role in lung cancer cells exposed to PAM is unknown. Here, we investigated the effect of *PRDX5* in PAM-treated A549 lung cancer cells and determined the mechanism underlying its cytotoxicity. Cell culture medium was treated with low temperature plasma at 16.4 kV for 0, 60, 120, or 180 s to develop PAM. *PRDX5* was knocked down in A549 cells via transfection with short hairpin RNA targeting *PRDX5*. Colony formation and wound healing assays, flow cytometry, fluorescence microscopy, and western blotting were performed to detect the effect of *PRDX5* knockdown on PAM-treated A549 cells. PAM showed higher cytotoxicity in lung cancer cells than in control cells, downregulated the mitogen-activated protein kinase signaling pathway, and induced apoptosis. *PRDX5* knockdown significantly inhibited cell colony formation and migration, increased ROS accumulation, and reduced mitochondrial membrane potential in lung cancer cells. Hence, *PRDX5* knockdown combined with PAM treatment represents an effective option for lung cancer treatment.

## INTRODUCTION

Approximately 1.8 million new cases of lung cancer and 1.2 million deaths associated with this disease are reported annually worldwide. Lung cancer represents the leading cause of cancer-related deaths globally and it is a serious threat to human health [1]. Primary malignant tumors of lung cancer can be classified into small cell lung cancer (SCLC) and non-small cell lung cancer (NSCLC); NSCLC is further classified into squamous cell carcinoma, adenocarcinoma, and large cell carcinoma [2]. NSCLC accounts for 80–90% of the total number of lung cancer cases and it has become the most common clinical malignant tumor. Fatal metastasis of NSCLC represents the most common cause of death in patients with lung cancer [3]. High proliferation and metastasis are associated with high mortality, and effective treatments remain unavailable. Therefore, it is necessary to develop new strategies to inhibit the proliferation and metastasis of lung cancer cells.

Plasma is the fourth state of matter along with solid, liquid, and gas. Non-thermal dielectric barrier discharge plasma (NTP) is a biologically active substance produced under high-voltage ionization conditions at atmospheric pressure, and it involves an electrode and a biological target. NTP shows application prospects in various research areas, including improving soybean [4] and sunflower [5] germination rates, optimizing chicken sperm motility [6], and promoting chicken embryo formation [7]. In medicine, NTP is used in cancer treatment [8] and clinical wound healing [9]. In cancer treatment, NTP can specifically kill oral squamous cell carcinoma cells based on the amount of catalytic Fe(II) present in the lysosome compared with that in fibroblasts. Lipid peroxidation, intracellular peroxide production, and superoxide production in mitochondria represent the main inducers of apoptosis [10]. Exposure to NTP leads to an increase in the level of reactive oxygen species (ROS) in the cell, which causes dose-dependent DNA damage and induces apoptosis in melanoma cells [10].

The selective killing of melanoma cells by NTP was first reported in 2007 [11] and it stimulated research on the potential of plasma in tumor therapy. In recent years, research on plasma-activated water or plasma-activated medium (PAM) has gained interest because of its availability, cost effectiveness, and good stability. PAM has been widely reported to show cytotoxicity in various cancer cells, including oral squamous cell carcinoma [10], colorectal cancer [12], breast cancer [13], and pancreatic cancer [14]. Normal cells maintain ROS balance, while tumor cell metabolism produces higher levels of ROS. Normal ROS levels regulate a series of cell activities but excessive ROS causes oxidative reduction, damage, and even apoptosis [15]. By producing high levels of ROS, such as hydrogen peroxide (H_2_O_2_), nitrate, and nitrite during ionization—all of which are dissolved in the medium—PAM shows a selective killing of cancer cells. Our previous studies have shown that PAM can induce autophagy in pancreatic cancer cells and induce apoptosis by inhibiting ROS-induced protein kinase B (AKT) and signal transducer and activator of transcription 3 signaling pathways, which can be reversed via treatment with N-acetyl cysteine (NAC); treatment with PAM alone also inhibits cell migration and invasion and colony formation [14].

Peroxiredoxins (PRDXs) are ubiquitous non-selenium peroxidases of approximately 22–27 kDa that can catalyze the reduction of peroxides and balance the redox state in cells [16–18]. PRDX5 is the only mammalian atypical 2-cysteine PRDX and it is located in mitochondria, peroxisomes, cytoplasm, and nuclei [19]. PRDX5 can scavenge ROS and reduce H_2_O_2_, alkyl hydroperoxide, and peroxynitrite levels [20], showing this antioxidant activity in the cytoplasm, mitochondria, and nuclei [21]. Research has shown that downregulation of *PRDX5* significantly increases ROS levels and apoptosis in cells exposed to ionizing radiation [22]. Our previous findings suggest that overexpression of *PRDX5* can eliminate intracellular ROS production and regulate the classical apoptotic pathway to inhibit emodin-induced apoptosis in gastric cancer cells [23]; *PRDX5* also mediates β-lapachone-induced apoptosis by reducing intracellular ROS levels and inhibiting the Wnt/β-catenin signaling pathway in SW480 colon cancer cells [24]. These findings imply that *PRDX5* may play a key role in the regulation of cancer cell apoptosis.

## RESULTS

### Effect of PAM treatment on A549 cells

**Figure 1:**
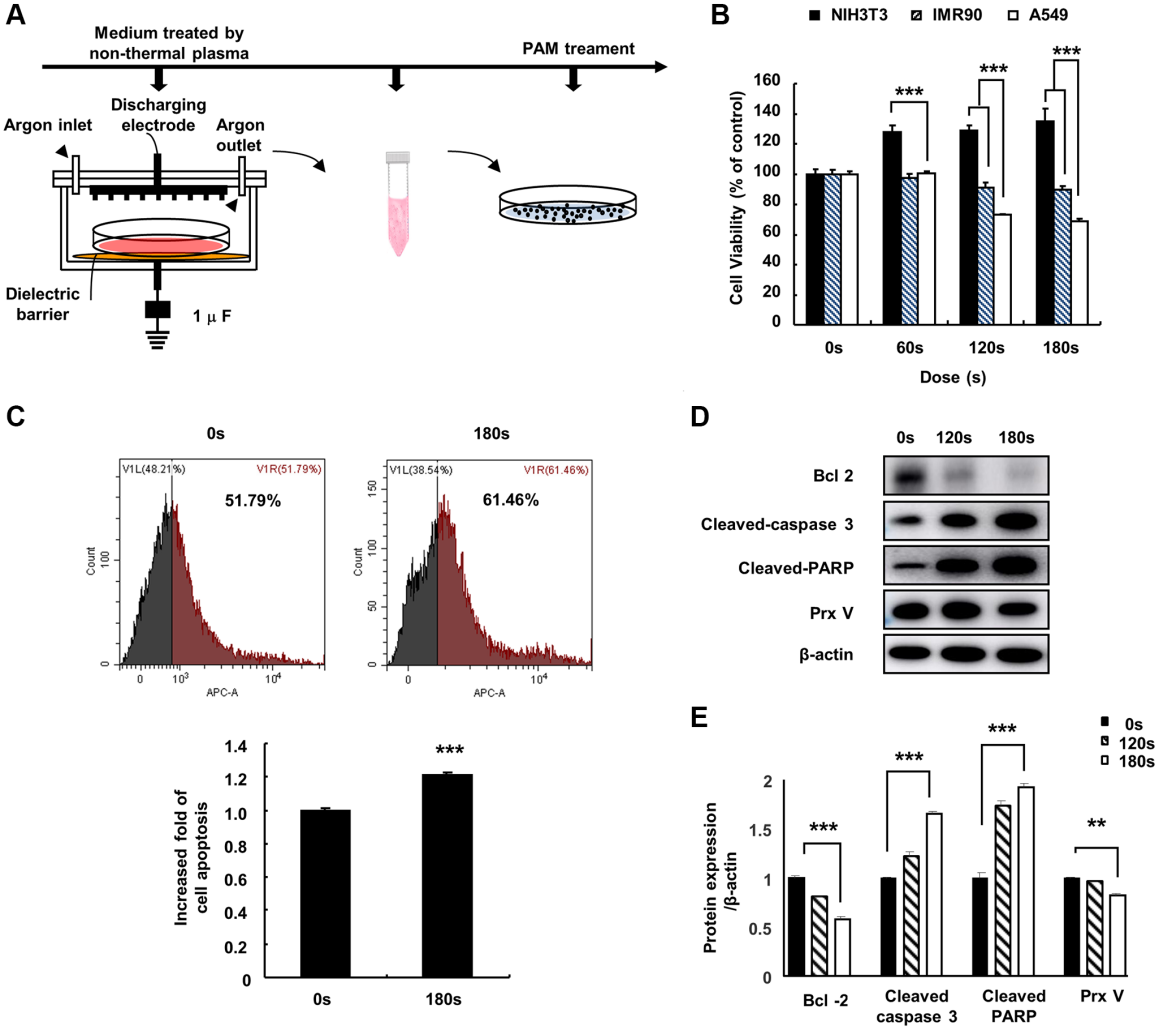
**Effect of PAM on A549 cells.** (**A**) Non-thermal PAM treatment experiment timeline. (**B**) The NIH3T3, IMR90, and A549 cells were treated with PAM (16.4 kV) in a dose-dependent manner for 24 h, and cell viability was measured using MTT assays. (**C**) Apoptotic A549 cells treated with PAM were detected via flow cytometry. (**D** and **E**): Western blotting of apoptosis-related proteins BCL2 apoptosis regulator, cleaved caspase-3, PARP, and PRDX5 in A549 cells after treatment with PAM (16.4 kV for 120 or 180 s). Quantified data are presented as the mean ± standard deviation of three independent experiments. Significant different are indicated at ^*^p < 0.05; ^**^p < 0.01; ^***^p < 0.001 vs. the control. Con, control.

To investigate the effect of PAM on lung cancer cell viability, we first treated the cell culture medium with low temperature plasma at 16.4 kV for 0, 60, 120, or 180 s to develop PAM (Figure 1A). The effects of PAM on the viability of NIH3T3 fibroblasts, IMR90 human embryonic fibroblasts, and A549 human NSCLC cells were examined using 3-(4,5-dimethylthiazol-2-yl)-2,5-diphenyl-2H-tetrazolium bromide (MTT) assays. PAM treatment inhibited the proliferation of A549 cells and did not show cytotoxicity in NIH3T3 and IMR90 cells (Figure 1B). Flow cytometry and western blotting were performed to further investigate the mechanism of PAM-mediated cytotoxicity; it was observed that the levels of cleaved caspase-3 and cleaved poly(ADP-ribose) polymerase (PARP) increased, while those of B cell lymphoma 2 (BCL2) and *PRDX5* decreased (Figure 1C–1E).

### PAM treatment induced ROS accumulation and mitochondrial membrane potential (MMP) loss in A549 cells leading to cell death

**Figure 2:**
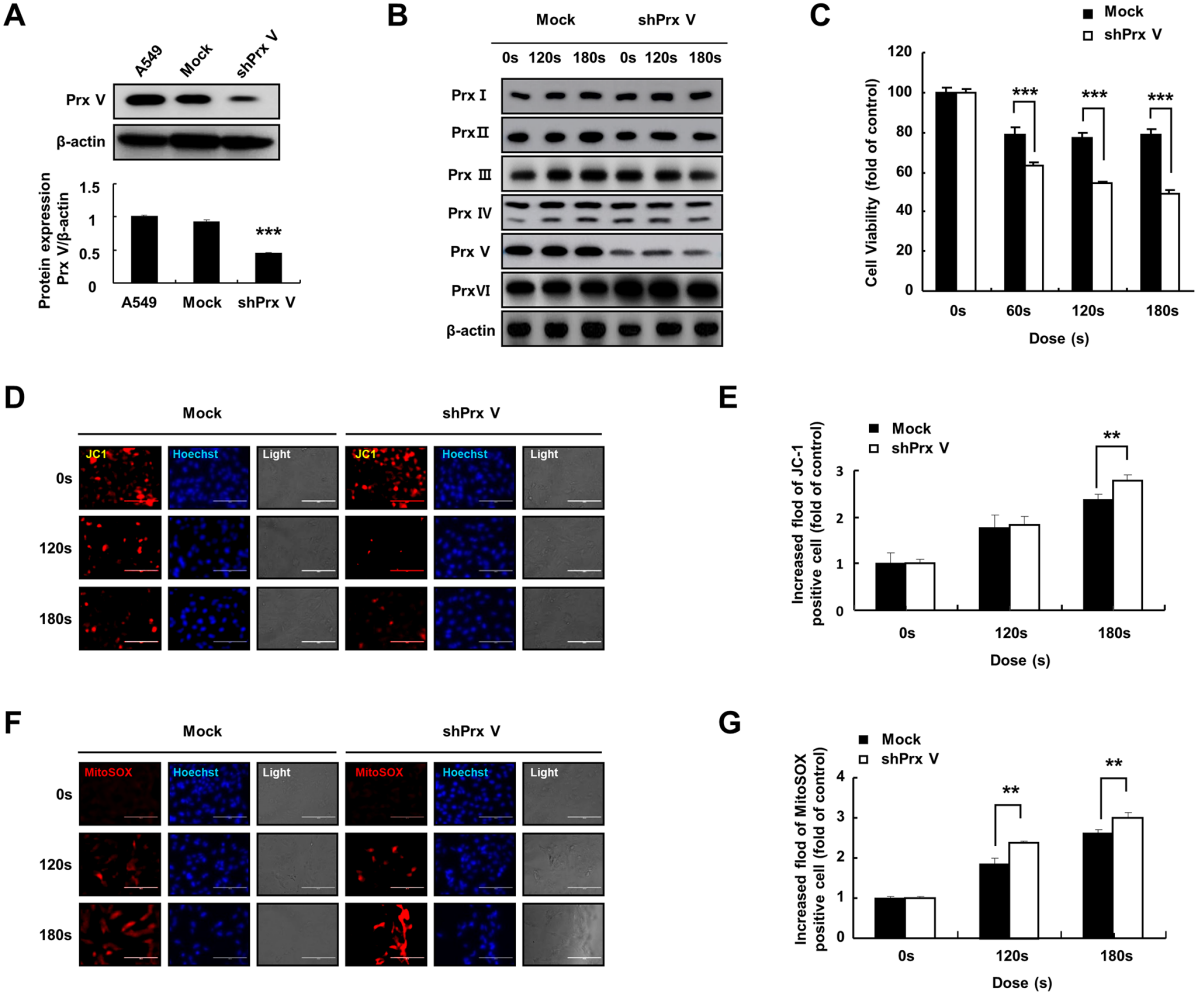
**PAM treatment can induce ROS accumulation and MMP loss in A549 cells leading to cell death.** (**A**) Knockdown of *PRDX5* after transfection with *shPRDX5* was verified in A549 cells via western blotting using PRDX5 antibodies. (**B**) Changes in expression levels of peroxide reductase family members in the A549 *PRDX5* knockdown cell line after PAM treatment. (**C**) Comparison of cell viability between the *shPRDX5* and control groups. (**D** and **E**) Imaging of *shPRDX5*-transfected A549 cells and control A549 cells exposed to PAM and stained with JC-1. (**F** and **G**) Relative fluorescence intensity of Mito-SOX staining. The data are presented as the mean ± standard deviation of three independent experiments. Significant differences are indicated at ^*^p < 0.05; ^**^p < 0.01; ^***^p < 0.001 vs. the control. Con, control.

To study the role of *PRDX5* in PAM-mediated induction of apoptosis in A549 lung cancer cells, we evaluated the suppression of *PRDX5* via western blotting using anti-*PRDX5* antibodies (Figure 2A). *PRDX5* expression was almost entirely suppressed in A549 cells transfected with small hairpin RNA (shRNA) targeting *PRDX5* (*shPRDX5*). As the previous experiment showed that *PRDX5* expression decreased in PAM-treated A549 lung cancer cells, we constructed a A549 *PRDX5* knockdown cell line, in which the expression level of *PRDX5* decreased after PAM treatment while the expression levels of the remaining *PRDXs* were maintained (Figure 2B). To investigate the potential regulatory effects of *PRDX5* on PAM-treated A549 lung cancer cells, MTT assays were performed, which showed that cell proliferation was significantly inhibited in the *PRDX5* knockdown group compared with that in the control group (Figure 2C). JC-1 fluorescence probe (Figure 2D and 2E) and Mito-SOX staining were used to detect changes in MMP, and it was found that apoptosis was enhanced and the MMP changed significantly in the *shPRDX5* group (Figure 2F and 2G).

### Knockdown of *PRDX5* enhanced the ability of PAM to inhibit the malignant behavior of A549 lung cancer cells

**Figure 3:**
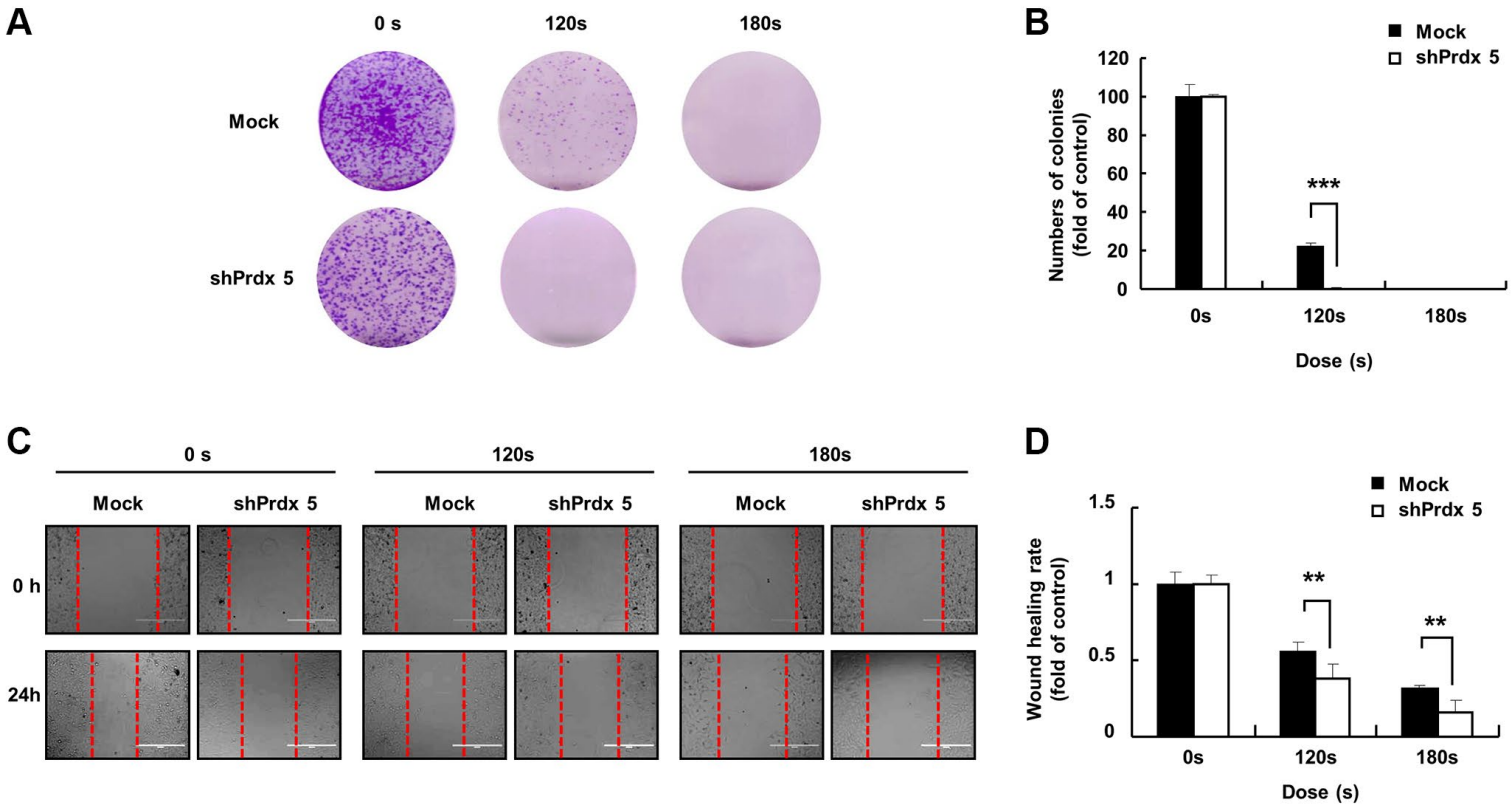
***PRDX5* knockdown enhanced the ability of PAM to inhibit the malignant behavior of A549 lung cancer cells.** (**A**) The day after cancer cells had attached to the bottom of plates and the medium was removed following PAM treatment. Upper panel: the colony-forming ability in A549 cells exposed to PAM for 7 days as detected via colony formation assays. (**B**) Quantification of colony formation data. (**C**) Images of the wound healing assay. (**D**) Quantification of wound healing area. The data are presented as the mean ± standard deviation of three independent experiments. Significant differences are indicated at ^*^p < 0.05; ^**^p < 0.01; ^***^p < 0.001 vs. the control. Con, control.

Malignant cancer cells are characterized by the potential for metastasis and proliferation [25]. Compared with the control group, the colony formation ability of the *shPRDX5* group was significantly inhibited after PAM treatment (Figure 3A and 3B). The wound-healing ability of *shPRDX5*-transfected A549 lung cells was reduced significantly (Figure 3C and 3D).

### *PRDX5* knockdown enhanced PAM-mediated inhibition of mitogen-activated protein kinase (MAPK) signaling pathway in A549 cells

**Figure 4:**
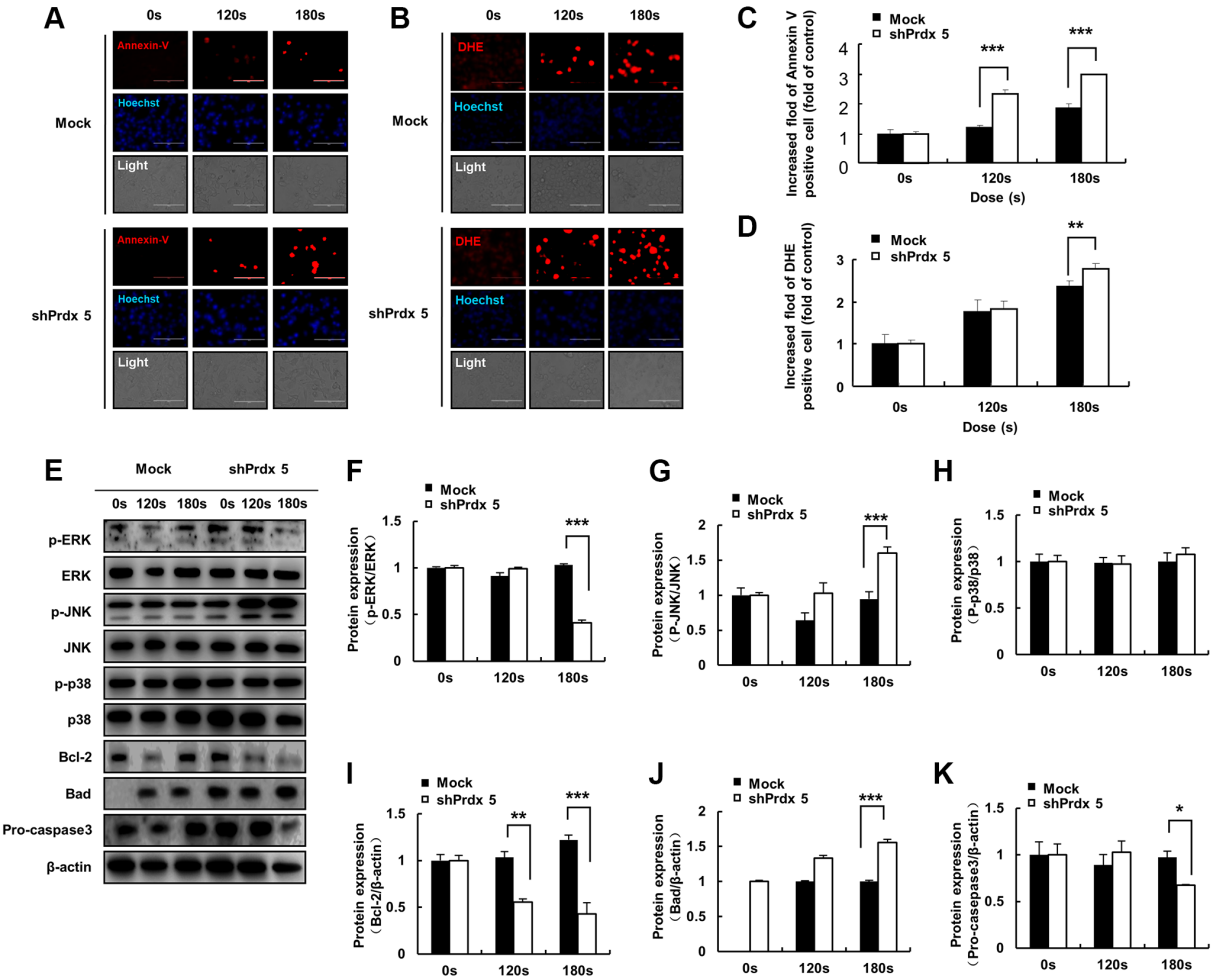
**PAM induces ROS accumulation and MMP loss in A549 cells, leading to cell death.** (**A**) Images of A549 cells exposed to PAM and stained with Annexin V. (**B**) Images of A549 cells exposed to PAM and stained with DHE. Relative fluorescence intensity of Annexin V staining. (**C**) Relative fluorescence intensity of Annexin V staining. (**D**) Relative fluorescence intensity of DHE staining. (**E**–**K**): Western blot analysis was performed to check the expression of apoptosis-related proteins. The data are presented as the mean ± standard deviation of three independent experiments. Significant differences are indicated at ^*^p < 0.05; ^**^p < 0.01; ^***^p < 0.001 vs. the control. Con, control.

In the previous experiment, the knockdown of *PRDX5* increased mitochondrial damage in A549 cells. To investigate the effect of PAM-induced ROS accumulation in A549 cells, we performed Annexin V and dihydroethidium (DHE) staining of cells treated with PAM to detect ROS generation and apoptosis. Knockdown of *PRDX5* enhanced apoptosis (Figure 4A and 4C) and superoxide levels (Figure 4B and 4D) in A549 cells. To characterize PAM-induced apoptosis after the knockdown of *PRDX5* in A549 lung cancer cells, we determined the expression levels of the important proteins of the MAPK pathway. We found that the expression levels of phosphorylated extracellular signal-regulated kinase (p-ERK)/ERK and BCL2 decreased. In contrast, the expression levels of phosphorylated c-Jun N-terminal kinase (p-JNK)/JNK, BCL2 associated agonist of cell death (BAD), and pro-caspase 3 were remarkably increased, while those of p38 were unchanged (Figure 4E–4K).

### NAC treatment reversed the inhibitory effect of PAM on the malignant behavior of A549 cells after *PRDX5* knockdown

**Figure 5:**
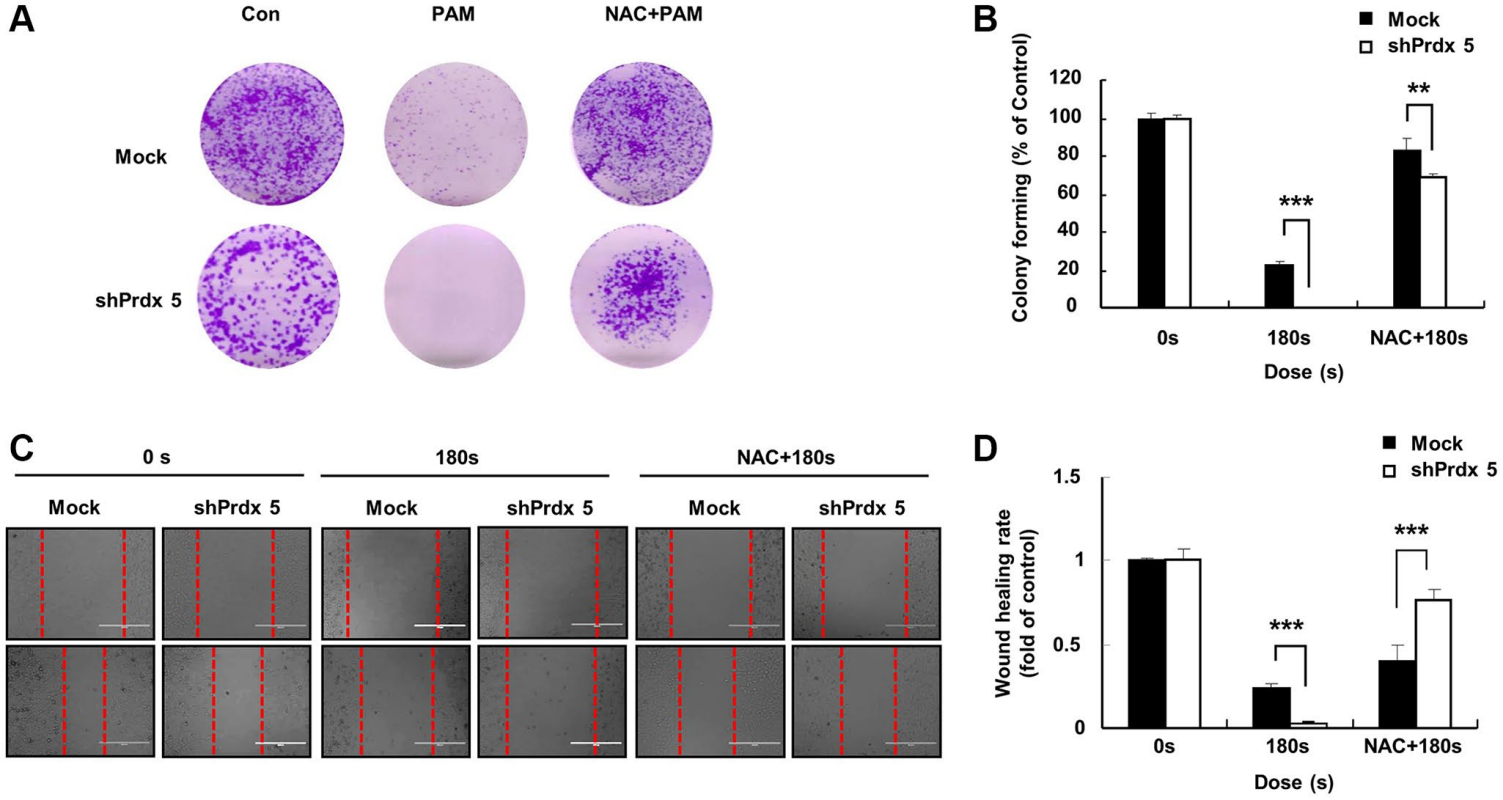
**NAC addition eliminated the inhibitory effect of PAM on the malignant behavior of A549 cells after *PRDX5* knockdown.** (**A**) Images on the first day after the cancer cells attached to the bottom of the plates; cells were pre-treated with NAC. The culture medium was removed, and PAM and NAC were added. (**B**) Colony formation assay performed to detect the colony formation ability of A549 cells after PAM exposure for 7 days. (**C**) Images of wound-healing assays. (**D)** Quantification of wound-healing area. The data are presented as the mean ± standard deviation of three independent experiments. Significant differences are indicated at ^*^p < 0.05; ^**^p < 0.01; ^***^p < 0.001 vs. the control. Con, control.

In a preliminarily experiment, we found that *PRDX5* knockdown induced ROS accumulation for oxidative stress generation. To further evaluate this effect, we added reactive NAC (a ROS scavenger) to pre-treated cells. NAC addition significantly restored the malignant behavior of A549 lung cancer cells, which was originally suppressed via PAM treatment. Compared with the control group, colony formation (Figure 5A and 5B) and wound healing (Figure 5C and 5D) abilities were significantly recovered in the *shPRDX5* group.

### NAC addition restored MMP

**Figure 6:**
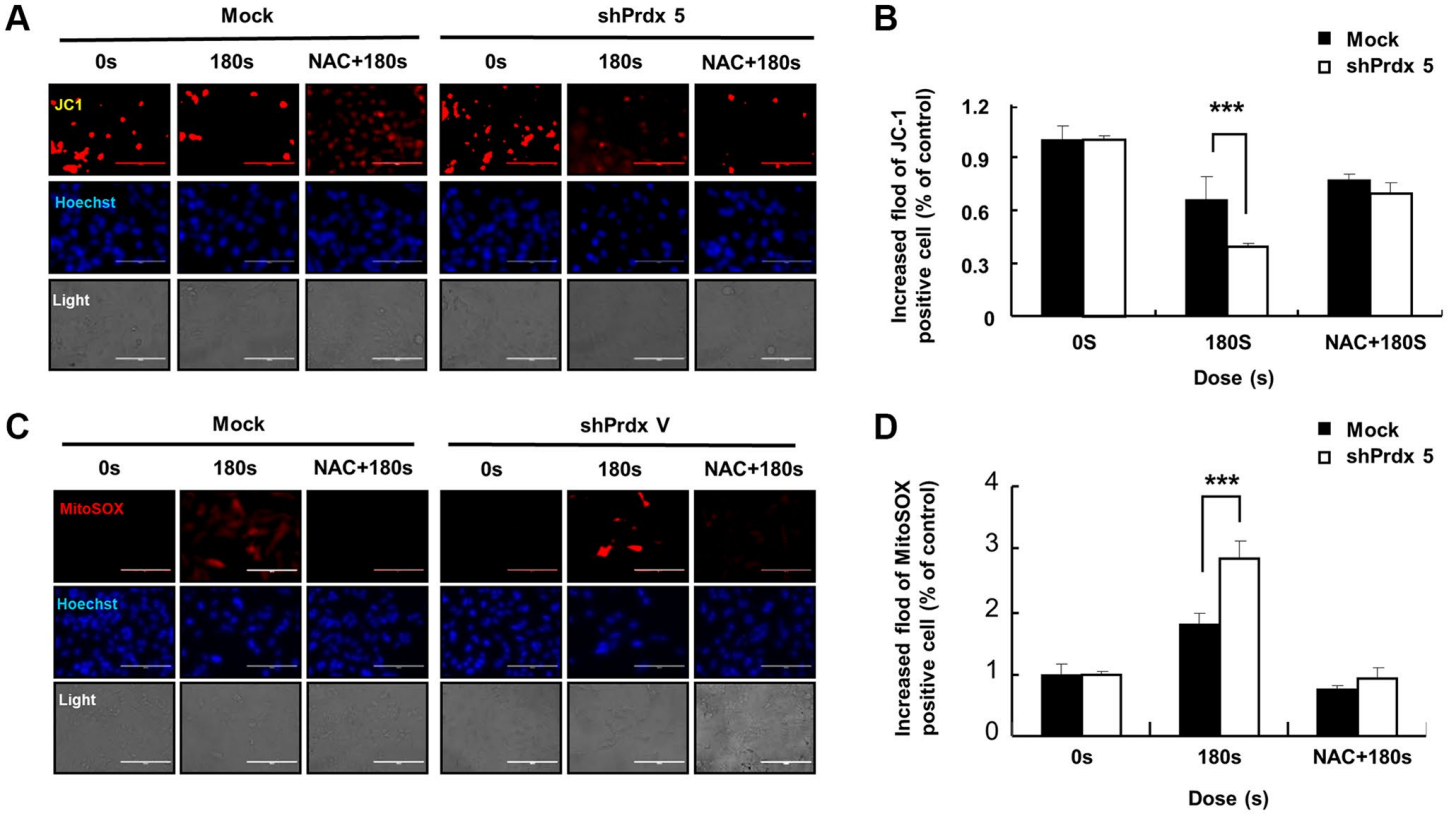
**The addition of NAC suppressed cell death and restored the MMP.** (**A** and **B**) Apoptotic A549 cells transfected with *shPRDX5* and Mock A549 cells were detected via Mito-SOX staining and observed under fluorescence microscopy after treatment with PAM. (**C** and **D**) Imaging of *shPRDX5*-transfected A549 cells and Mock A549 cells exposed to PAM and stained with JC-1. The data are presented as the mean ± standard deviation of three independent experiments. Significant differences are indicated at ^*^p < 0.05; ^**^p < 0.01; ^***^p < 0.001 vs. the control. Con, control.

We found that NAC addition slowed down the inhibitory effect of PAM on the malignant behavior of A549 cells. Therefore, we further explored the effects of NAC addition on cell apoptosis and MMP. After pre-treatment with NAC, Mito-SOX staining and the JC-1 fluorescence probe were used to evaluate cell death and MMP in A549 cells. NAC addition resulted in the recovery of MMP (Figure 6A and 6B) and a significant suppression of apoptosis in the *shPRDX5* group compared with that in the control group (Figure 6C and 6D).

### Effects of NAC treatment on intracellular ROS levels in *PRDX5* knockdown A549 cells

**Figure 7:**
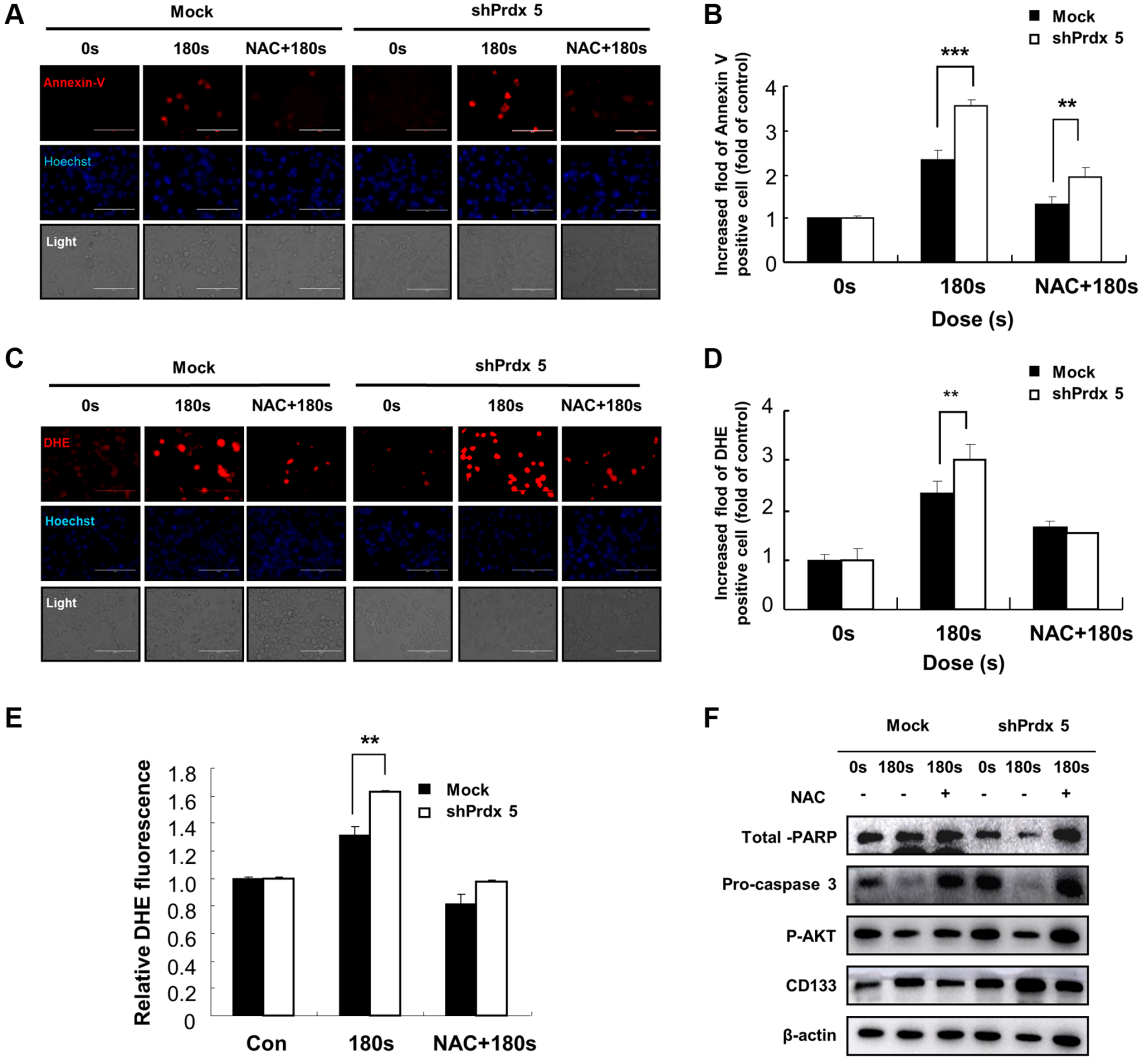
**Effects of NAC treatment on intracellular ROS content in *PRDX5* knockdown A549 lung cancer cells.** (**A**) Images of A549 cells exposed to PAM and stained with Annexin V after NAC treatment. (**B**) Relative fluorescence intensity of Annexin V staining. (**C**) Images of A549 cells exposed to PAM and stained with DHE after NAC treatment. (**D**) Relative fluorescence intensity of DHE staining. (**E**) Flow cytometric analysis of intracellular ROS production in A549 cells using DHE staining. (**F**) Western blotting of apoptosis-related proteins, including AKT, pro-caspase 3, and PARP in A549 cells after treatment with NAC. Quantified data are presented as the mean ± standard deviation of three independent experiments. Significant differences are indicated at ^*^p < 0.05; ^**^p < 0.01; ^***^p < 0.001 vs. the control. Con, control.

To prove that apoptosis in A549 cells mediated by increased ROS level is caused by the knockdown of *PRDX5*, we pre-treated *shPRDX5-*transfected cells with NAC. Annexin V staining showed that cell apoptosis was suppressed after NAC addition and had almost returned to the original level (Figure 7A and 7B). However, compared with the control group, the changes in superoxide levels in the *shPRDX5* group were more obvious. Moreover, the DHE fluorescence probe was used to detect changes in intracellular ROS levels, and those in the *shPRDX5* group varied significantly (Figure 7C and 7D). Flow cytometry analysis showed similar results (Figure 7E). For quantitative evaluation, the levels of AKT, PARP, and caspase involved in apoptosis were detected via western blotting. PAM treatment significantly increased the levels of phosphorylated AKT, PARP, and caspase; however, this was not observed in NAC-treated cells (Figure 7F).

### *PRDX5* maintains the mitochondrial state and regulates MAPK signaling against PAM-induced apoptosis

**Figure 8:**
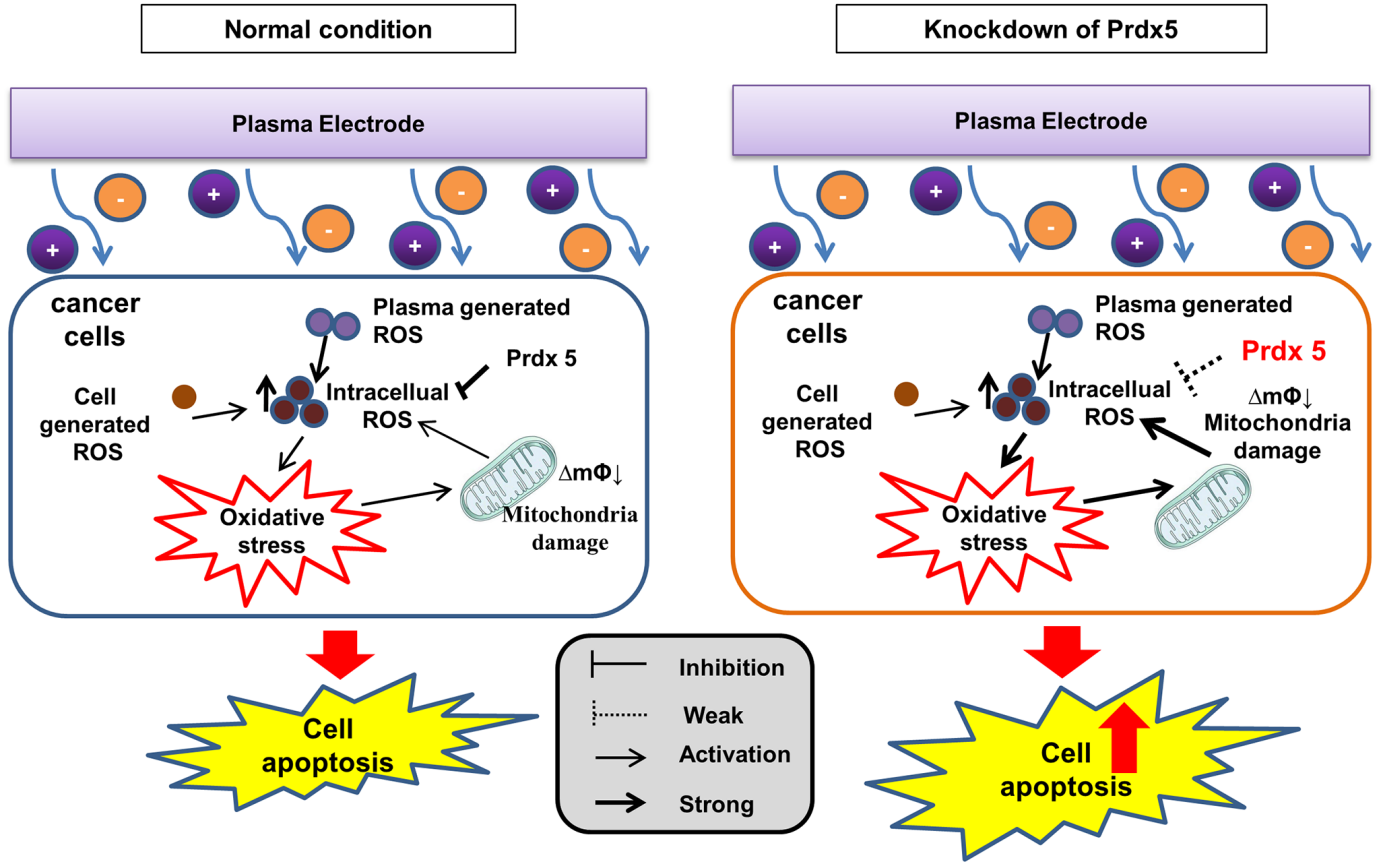
PRDX5 maintains the mitochondrial state and regulates MAPK signaling against PAM-induced apoptosis.

When plasma comes into contact with a biological target through PAM, plasma-generated ROS are the major contributors to cell death. The ROS produced within cells and the large accumulation of ROS in the low-temperature plasma cause damage to the cell oxidation/reduction system. This affects the mitochondrial state, leading to a decrease in the MMP. As a regulator of cell growth, the MAPK signaling pathway associated with tumor survival leads to apoptosis. By removing ROS, *PRDX5* can relieve PAM-induced apoptosis (Figure 8).

## DISCUSSION

The specific anticancer mechanism of NTP *in vivo* is still an open question, although several studies have indicated the large amount of ROS produced by NTP plays an important role. A significant increase in the levels of intracellular ROS is the most common observation in NTP-treated cells. The level of endogenous ROS in cancer cells, especially p53-deficient cells, is higher than that in normal cells due to the overactivation of oncogenes and abnormal metabolism [26]. Therefore, cancer cells must survive in a higher oxidative stress environment and are more vulnerable to ROS. Excessive ROS induces oxidative stress, directly attacks DNA, proteins, lipids, and other cellular components, activates the MAPK signaling pathway, and leads to apoptosis [27]. Treatment options for lung cancer are limited due to its enhanced metastatic phenotype and chemotherapy tolerance [28]. NTP treatment is regarded as an innovative anticancer approach that induces apoptosis, autophagy, and immunogenic cell death by increasing intratumoral ROS levels [29]. Tumor cells express high levels of antioxidant proteins to reduce ROS levels and reestablish redox balance while maintaining pro-tumorigenic signaling and resistance to apoptosis. The altered redox balance of tumor cells compared to normal cells identifies ROS manipulation as a potential target for cancer therapy [30]. The use of an appropriate conditional PAM for its lethal effect on lung cancer cells is considered reasonable, and this effect was confirmed via *PRDX5* knockdown. PAM treatment showed higher toxicity in tumor cells than in normal cells (Figure 1) and it effectively inhibited A549 cell survival (Figure 2A), which may be attributed to changes in the MMP (Figure 2D). PAM treatment increased the accumulation of mitochondrial and total ROS in cells (Figure 2B and 2E), increased the levels of apoptosis-related proteins (Figure 4E), and enhanced apoptosis (Figure 4A). Moreover, we confirmed that the MAPK signaling pathway was associated with PAM-induced apoptosis in A549 cells (Figure 4E–4K). After NAC addition, we found that the accumulation of mitochondrial and cellular ROS (Figures 6C and 7C) and cell death caused by PAM (Figures 6A and 7A) were suppressed. Furthermore, colony formation and migration were recovered in A549 cells after NAC addition (Figure 5).

Our data showed that *PRDX5* knockdown and PAM treatment induced ROS generation, downregulated the anti-apoptotic protein BCL2, disrupted the MMP, and enhanced apoptosis in A549 lung cancer cells. PRDXs are a family of thiol peroxidases that play a key role in maintaining the redox state. The *PRDX* gene family has six subtypes (*PRDX 1–6*), of which *PRDX1, PRDX2,* and *PRDX6* are mainly expressed in the cytoplasm; *PRDX4* is expressed in the endoplasmic reticulum while *PRDX3* and *PRDX5* are expressed in the mitochondria. The lung is a crucial organ that regulates whole-body gas exchange; it shows high energy metabolism and contains a large number of mitochondria. Lung diseases are caused by several factors, including ROS, which are closely associated with *PRDX5* function. PAM treatment produces a large amount of ROS, leading to an imbalance in the oxidation/reduction system of cancer cells, which causes oxidative stress, mitochondrial dysfunction, and ultimately cell death. Therefore, *PRDX5* expression is likely to be regulated during apoptosis in A549 lung cancer cells treated with PAM. In the present study, *PRDX5* knockdown enhanced the effect of PAM on A549 lung cancer cells via the MAPK signaling pathway (Figure 4E and 4F). Notably, a decrease in the expression levels of p-ERK/ERK was observed while the expression levels of p-JNK/JNK were remarkably increased. However, p38 expression levels did not change. ERK signaling is related to the growth and differentiation of cells, JNK signaling is related to apoptosis and tumor survival, and p38 plays a role in inflammatory response. The present results showed that PAM treatment suppressed the proliferation of *shPRDX5*-transfected A549 cells and enhanced apoptosis. Although PAM treatment may not cause inflammation in A549 lung cancer cells, the changes induced on ERK and JNK signaling pathways protein levels may inhibit p38 expression. To investigate this further, a MAPK inhibitor should be used in future studies. The parallel MAPK signaling pathway has different characteristics and biological effects on different cells, and the duration of its activation also varies. Moreover, as the whole process of interclass signaling differs from that of MAPK, the coordination of the several signaling pathways can produce different, and even opposite biological effects. This should also be considered in future research on the effects of PAM in MAPK signaling.

Cancer stem cells (CSCs) exhibit the potential for self-renewal, differentiation, and tumorigenicity. They represent the major cause of cancer therapy failure because of certain mechanisms that confer considerable resistance to chemotherapy and radiotherapy [31]. Low levels of ROS induce the differentiation of CSCs [32]. The redox balance in CSCs represents an important target for tumor therapy [33]. PAM treatment produces ROS, which may inhibit CSC growth. PAM mainly comprises ROS such as H_2_O_2_, superoxide, singlet oxygen, and hydroxyl radicals, among others [34]. It has been established that high levels of ROS can damage malignant cells [35]. Currently, chemotherapeutic drugs generate oxidative stress by increasing intracellular ROS production, which damages the structure of DNA and proteins and causes mitochondrial damage and even apoptosis [36]. PAM can kill cells by producing a large amount of intracellular ROS and *PRDX5* function is also associated with intracellular ROS production; however, the role of *PRDX5* in regulating PAM-induced cell death has not been fully studied. In the present study, *PRDX5* knockdown induced an increase in intracellular ROS levels, which was restored via treatment with a ROS scavenger, NAC. The CD133 protein level has been reported to decrease after *PRDX5* knockdown. CD133 is a specific marker molecule expressed independently on the surface of tumor stem cells in various tissues [37], and it is associated with tumor self-renewal, differentiation potential, signal transduction, drug tolerance, recurrence, and prognosis [38]. The present results suggest that the regulation of *PRDX5* function may represent a new therapeutic alternative for the treatment of CSCs in NSCLCs. To increase the potential of *PRDX5* as a therapeutic target in CSCs, the regulatory mechanism of *PRDX5* in PAM-treated cancer cells should be studied further, as the present results provide preliminary data for developing new treatment options. Based on the findings of this study, the regulation of *PRDX5* in combination with PAM treatment in the treatment of lung CSCs should be investigated in future studies.

## MATERIALS AND METHODS

### Cell culture and media

A549 human NSCLC cells, NIH3T3 mouse embryo fibroblasts, and IMR90 human lung embryo fibroblasts were obtained from the American Type Culture Collection (Manassas, VA, USA). A549 and IMR90 cells were cultured in Dulbecco’s modified Eagle’s medium (DMEM; Gibco, Waltham, MA, USA) supplemented with 10% fetal bovine serum (FBS) and 1% penicillin–streptomycin liquid (Solarbio Life Sciences, Beijing, China). The cells were maintained at 37°C in a humidified incubator with 5% CO_2_.

### PAM treatment and NAC treatment

To generate PAM, 10 mL cell culture medium samples in 100 mm Petri dishes were exposed to 16.4 kV NTP (Figure 1A) for different periods (0, 60, 120, or 180 s). The obtained PAM were then filtered through 0.22 μm filters before use. All PAM were produced under the same experimental conditions (gas flow at 3 L/min, exposure frequency, applied voltage, and pulse duration), and the same distance was maintained between the tip of electrode needles and the upper surface of the medium (20 mm). Fresh PAM were prepared before each experiment. In the NAC co-treatment group, 2.5 mM of NAC was added to culture medium after PAM treatment.

### Cell viability assay

MTT assays were performed to evaluate cell viability. Cells were seeded at a density of 1 × 10^4^ cells/well in 96-well plates and treated with the different PAM for 24 h (37°C and 5% CO_2_). The accumulation of formazan (dissolved in dimethyl sulfoxide) was determined following the addition of MTT reagent (5 mg/mL), and the absorbance was measured at a wavelength of 490 nm using the UV max Kinetic microplate reader (Molecular Devices, Sunnyvale, CA, USA).

### Cell apoptosis assay

Cells were suspended in apoptosis stain buffer and then stained with Annexin V-Phycoerythrin and Hoechst (PE) using an apoptosis detection kit (BD Biosciences, Franklin Lakes, NJ, USA) according to the manufacturer’s protocol. The cells were then washed with phosphate-buffered saline (PBS) and Annexin-PE-positive cells were identified via fluorescence microscopy and flow cytometry on a BD FACSCalibur instrument (BD Biosciences). The images were analyzed using WinMDI software (version 2.9; BD Biosciences).

### Western blotting

Cell proteins were lysed in lysis buffer and separated on 12% sodium dodecyl sulfate-polyacrylamide gels at a concentration of 20 μg, and then transferred onto nitrocellulose membranes (Millipore, Bedford, MA, USA). The membranes were blotted with primary antibodies against PRDX5, BCL2, BAD, caspase 3, JNK, p-JNK, ERK, p-ERK, p38, p-p38 (all purchased from Santa Cruz Biotechnology, Dallas, TX, USA), and β-actin (Sigma–Aldrich, St. Louis, MO, USA) at 4°C for 6 h after blocking with skimmed milk for 1 h. The membranes were washed five times with 10 mM Tris-HCl (pH 7.5) containing 150 mM NaCl (Tris-buffered saline, TBS) and 0.2% Tween 20, and subsequently incubated with horseradish peroxidase-conjugated goat anti-rabbit IgG or anti-mouse IgG (both from Sigma–Aldrich) for 1 h at room temperature. After the removal of excess antibodies by washing with TBS, specific binding was detected using a chemiluminescence detection system (Amersham, Berkshire, UK) according to the manufacturer's instructions.

### Construction of stable *PRDX5*-knockdown A549 cells

The shRNA specific to *PRDX5* (*shPRDX5* LV3, H1&Puro) and control shRNA LV3 (H1&Puro) lentivirus vectors used for the knockdown experiment were purchased from Shanghai GenePharma (Shanghai, China). The targeted sequence of s*hPRDX5* was 5′-GGAATCGACGTCTCAAGAGGT-3′ and the targeted sequence of the negative control was 5′-GTTCTCCGAACGTGTCACGT-3′. A549 cells (1 × 10^4^/well) were seeded in a 96-well cell culture plate for 24 h (37°C and 5% CO_2_) prior to transfection. The culture medium was first replaced with polybrene (5 μg/mL; Shanghai GenePharma) and packed lentivirus with a multiplicity of infection for 48 h, and then changed to complete culture medium (DMEM supplemented with 10% FBS and antibiotics). Infected cells were selected via treatment with puromycin. Western blotting was performed to analyze the expression levels of PRDX5.

### Cellular and mitochondrial ROS detection

DHE was used as a fluorescent probe to determine changes in intracellular ROS levels. PAM-treated and control A549 cells were collected and washed following incubation with DHE. Mitochondrial ROS levels were detected using Mito-SOX Red (Thermo Fisher Scientific, Waltham, MA, USA). Fluorescence microscopy and flow cytometry were performed to analyze cells.

### Cell MMP analysis

The cell MMP was detected using the JC-1 probe (Sigma–Aldrich). PAM-treated and control A549 cells were washed and stained with JC-1. A fluorescence microscope was used to obtain images of the A549 cells. Fluorescence intensities were analyzed using ImageJ software (National Institutes of Health, Bethesda, MD, USA).

### Colony formation assay

A549 cells (1 × 10^3^ cells/well) were seeded in six-well plates, treated with PAM (16.4 kV for 0 s, 120 s, or 180 s) for 24 h, and maintained at 37°C in a 5% CO_2_ incubator for 7 days. The cells were then washed with PBS, fixed with 3.7% formaldehyde for 10 min, treated with methanol for 20 min, and stained with 0.05% crystal violet for 30 min. The plates were washed with PBS thrice prior to obtaining images. Colony formation was calculated as the number of colonies formed under treatment/number of colonies formed in the control group.

### Wound healing assay

A549 cells were seeded onto IncuCyte ImageLock 48-well microplates (Sartorius AG, Göttingen, Germany), at a density of 2 × 10^6^ cells/well without PAM. A linear scratch was performed in the cell monolayer using a wound maker (Sigma-Aldrich). The cells were rinsed with 1× PBS thrice, and PAM (16.4 kV for 0 s, 120 s, or 180 s) was added. Photomicrographs of the cells were taken at 0 and 24 h and processed using ImageJ (National Institutes of Health). The wound healing area was calculated as the scratch area in the treatment group/scratch area in the control group × 100%.

### Statistical analysis

Data are presented as the mean ± standard deviation of three independent experiments. Two-way ANOVA was performed to determine significant differences (*p* < 0.05) among treatment groups. Statistical analysis was performed using the Statistical Package for the Social Sciences (SPSS, version 19.0; IBM, Armonk, NY, USA).
